# Quantum nanophotonics

**DOI:** 10.1515/nanoph-2023-0059

**Published:** 2023-02-10

**Authors:** Jaehyuck Jang, Minsu Jeong, Junsuk Rho

**Affiliations:** Department of Chemical Engineering, Pohang University of Science and Technology (POSTECH), Pohang 37673, Republic of Korea; Department of Mechanical Engineering, Pohang University of Science and Technology (POSTECH), Pohang, 37673, Republic of Korea; POSCO-POSTECH-RIST Convergence Research Center for Flat Optics and Metaphotonics, Pohang 37673, Republic of Korea

In recent years, the field of quantum nanophotonics has been thriving with an intense interest in the development of new theories, devices, and applications for specific uses. This special issue on “Quantum nanophotonics” highlights some recent developments in this topic through reviews, perspectives, and research papers.

This issue contains review and perspective articles that provide a comprehensive overview of cutting-edge topics. Chang and Zwiller [1] review the recent progresses in integrated quantum photonics using nanowires, focusing on integrated emitters, detectors, and fabrication methods. This review also presents the applications for quantum information processing and sensing based on nanowires. Gali [2] summarizes the ab initio theory to fully understand solid-state qubits, which are an essential component in the quantum photonic device. This computational method has been applied to the calculation of excited states, photo-ionization threshold, optical excitation spectra, effective mass states, and spin dynamics. This approach could give insights beyond conventional density functional theory that cannot fully capture the characteristics of excited states.

Biotechnologies are being used in a variety of quantum optics and photonics or vice versa. DNA nanotechnology, using the information of DNA to design and manufacture artificial nucleic acid structures for technological uses, has been utilized in the field of quantum emitters. Exotic traits of DNA enable the grabbing of quantum emitters and steering of their directors at the molecular level of resolution. The relevant studies were extensively reviewed by Cho et al. [3]. Reversely, the understanding of light–matter interaction in quantum optics provides a hint to investigate chemical and biological processes. Kim et al. [4] present a review of plexcitonic and vibro-polaritonic strong coupling based on light–matter interaction with optical resonators. The authors introduce from fundamentals of strong coupling, configuration and characteristics of plexcitons, and applications for chemistry and biological detection.

Kim et al. [5] discuss that nanophotonic resonance engineering can make the near-unity readout fidelity necessary for improving the sensitivity of NV quantum sensing. The perspective gives insight into the background of NV quantum sensing, applications of resonant structures, and the remaining challenges for practical sensing. Zheng and Kim [6] address the degradation mechanisms of perovskite-based light-emitting diodes. The degradation can potentially occur in external and internal processes, leading to distinct effects on performance and stability.

Various research articles on quantum nanophotonics are included. There is growing interest in optimizing the single photon emitter (SPE) that can be integrated into the photonic circuit and enable practical application. Azzam et al. [7] demonstrate the Purcell enhancement of WSe_2_ SPE using a dielectric cavity. The dielectric cavity imposes an oriented strain profile on the WSe_2_, which can make the polarization state of the SPE selectively controlled. Xu et al. [8] report a high-purity SPE based on nanodiamonds (NDs) with negatively charged silicon vacancy (SiV^−^) centers using an ion implantation method. They successfully prevent SiV^−^ emitters from being created in clusters while overcoming several problems of ion implantation into NDs, such as extremely small implantation cross sections and high susceptibility to ion beam damage. Single photon detection is the major branch of integrated quantum photonics. Chae et al. [9] propose a tunable up-conversion single photon detector that can operate at the complete telecom C band using the type-0 sum-frequency generation process. The broad detection range is obtained from a tunable optical parametric oscillator pump laser satisfying the phase-matching condition of the harmonic generation. Vetlugin et al. [10] report a method for photon number resolving detection making a superconducting nanowire single photon detector array coherently absorb a single optical mode. This method being able to replace the conventional optical mode multiplication can effectively discriminate an arbitrary photon number. Research for quantum photonic-integrated circuits is intensely conducted to improve quantum technology applications. Lu et al. [11] proposed a rod and a slit photonic crystal microring cavity with a high-quality factor (>10^6^), preserving the waveguide coupling property of the standard whispering gallery mode. Being able to alternate the conventional microgear photonic crystal ring whose etched surface is too close to potential quantum emitters, this cavity can be a promising platform for cavity quantum electrodynamics. A waveguide designed for spontaneous four-wave mixing makes an integrated photonic system generate a quantum light source itself. However, even at the propagation loss on the photon pair through the waveguide simultaneously exists, research for the loss had not been actively conducted. Shin et al. [12] investigate the propagation loss effects on the photon-pair generation by conducting both theoretical and experimental demonstrations.

Advanced quantum optics theories, which stem from classical optics, are discussed in this issue as well. Vázquez-Lozano and Liberal [13] report how the anisotropic temporal boundaries can control the angular properties of the vacuum amplification effect. The authors provide several examples for the angular distribution of generated photons: inhibition of photon production along a specific direction, resonantly directive amplification, generation of angular combs, and fast variation between inhibition and resonant amplification. Chao et al. [14] report an improved framework for assessing the maximum spectral-integrated local density of states (LDOS) being able to obtain in a structured media. This article analytically derives the scaling law between the upper bound of LDOS, material susceptibility, and source bandwidth. Feng et al. [15] introduce quantum percolation and provide its experimental demonstration using prototyped waveguide lattices. The waveguide lattices with different occupation probabilities were fabricated as on-chip configurations using the direct laser writing technique. This approach to directly observe the quantum transport phenomena would be useful for statistical exploration in future studies on quantum percolation dynamics.

Metasurface, which has more degrees of freedom to manipulate photons than traditional optical elements, can provide opportunities for novel quantum measurements. Gao et al. [16] demonstrate that a metasurface enables fully distinguishing all four polarization bell states without additional degrees of freedom or assisting auxiliary photons. The metasurface, which allows controlling the different polarization in two output channels, realizes the deterministic measurement of bell states. Yung et al. [17] demonstrate metasurface-driven jones-matrix profile imaging for an unknown object applying two-photon interference. The unknown polarization response of an object can be retrieved by coincidence measurement of known polarization response from reference metasurface. Quantum metasurface, in which the classical concept of metasurface and quantum effects are combined, can emerge exotic phenomenon. Nefedkin et al. [18] demonstrate that the large nonreciprocity in free space can be observed from the quantum metasurface formed by periodic arrays of two-level atoms by using nonlinearity. The total cross section shows distinct differences depending on the direction of the input wave by engineering the position of atoms and detuning the transition frequencies.

Lastly, next-generation nanophotonic devices are introduced. Zhang et al. [19] introduce palladium diselenide photodetectors with asymmetric van der Waals contact that support self-powered photodetection. The optoelectronic properties of palladium diselenide ensure a broadband photoresponse in the visible-NIR regime and polarization-sensitive absorption. The authors further provide polarimetric imaging using the proposed photodetectors. In addition, various analytical techniques for evaluating perovskite light-emitting diodes were introduced. Jeong et al. [20] proposed a plasmonic-amplified third harmonic generator. The authors utilize a plasmonic nanoantenna array to improve the beam stability of position-dependent third-harmonic yield. This method could be useful in applications such as wavefront-shaping nonlinear metasurfaces and highly sensitive biosensors. Shorter et al. [21] investigate dye-doped polymer on metallic substrates and observe its anomalous reflection dips in the ultraviolet regime. The detailed analysis of this peculiar phenomenon is provided with the concept of surface plasmons and their singularity.

In sum, we hope that this collection of articles offers an extensive overview of the rapidly growing field of quantum nanophotonics and will inspire further research and diverse applications. We would like to appreciate all the authors and reviewers for their contributions.
